# Statistical significance approximation in local trend analysis of high-throughput time-series data using the theory of Markov chains

**DOI:** 10.1186/s12859-015-0732-8

**Published:** 2015-09-21

**Authors:** Li C. Xia, Dongmei Ai, Jacob A. Cram, Xiaoyi Liang, Jed A. Fuhrman, Fengzhu Sun

**Affiliations:** 1 Department of Medicine, Division of Oncology, Stanford University School of Medicine, Stanford, 94305-5151 CA USA; 2 Department of Statistics, The Wharton School, University of Pennsylvania, Philadelphia, 19104 PA USA; 3 School of Mathematics and Physics, University of Science and Technology Beijing, Beijing, 100083 China; 4 Marine and Environmental Biology, Department of Biological Sciences, University of Southern California, Los Angeles, 90089-0371 CA USA; 5 Molecular and Computational Biology, Department of Biological Sciences, University of Southern California, Los Angeles, 90089-2910 CA USA; 6 Centre for Computational Systems Biology, Fudan University, Shanghai, 200433 China

## Abstract

**Background:**

Local trend (i.e. shape) analysis of time series data reveals co-changing patterns in dynamics of biological systems. However, slow permutation procedures to evaluate the statistical significance of local trend scores have limited its applications to high-throughput time series data analysis, *e*.*g*., data from the next generation sequencing technology based studies.

**Results:**

By extending the theories for the tail probability of the range of sum of Markovian random variables, we propose formulae for approximating the statistical significance of local trend scores. Using simulations and real data, we show that the approximate p-value is close to that obtained using a large number of permutations (starting at time points >20 with no delay and >30 with delay of at most three time steps) in that the non-zero decimals of the p-values obtained by the approximation and the permutations are mostly the same when the approximate p-value is less than 0.05. In addition, the approximate p-value is slightly larger than that based on permutations making hypothesis testing based on the approximate p-value conservative. The approximation enables efficient calculation of *p*-values for pairwise local trend analysis, making large scale all-versus-all comparisons possible. We also propose a hybrid approach by integrating the approximation and permutations to obtain accurate p-values for significantly associated pairs. We further demonstrate its use with the analysis of the Polymouth Marine Laboratory (PML) microbial community time series from high-throughput sequencing data and found interesting organism co-occurrence dynamic patterns.

**Availability:**

The software tool is integrated into the eLSA software package that now provides accelerated local trend and similarity analysis pipelines for time series data. The package is freely available from the eLSA website: http://bitbucket.org/charade/elsa.

**Electronic supplementary material:**

The online version of this article (doi:10.1186/s12859-015-0732-8) contains supplementary material, which is available to authorized users.

## Background

Time series data are important resources to explore the dynamics of biological systems, where the factors of interest could be genes in gene regulation studies, or organisms and/or environmental factors in ecological studies. Identifying reliable association patterns between these factors could further our understanding of the functionality and interaction of biological systems [1, 2]. When the actual associations are active only within certain time subintervals or the responses lag the stimulants [3, 4], ordinary correlation based analysis methods (i.e. Pearson’s and Spearman’s correlation) considering the expression/abundance profiles across the entire time span may fail to recover these local and potentially time-delayed association patterns. Fortunately, a wealth of computational methods had been developed to overcome such difficulties, such as local similarity analysis [3, 5, 6] and local trend (shape) analysis [4, 7]. Those methods complement ordinary analytical approaches and have important applications in gene profile clustering, regulatory network construction, co-occurrence pattern identification and many other areas [3–9]. For instance, Qian et al. [3] proposed a local similarity based measure to identify local and potential time-delayed associations between gene expression profiles. This local similarity analysis technique is further extended and successfully applied to microbial ecology time series studies [5, 6, 10, 11].

In local similarity analysis, *local* indicates the two factors are only associated within some time subinterval, and *time-delayed* indicates there is a time shift in the associated profiles. The strength of the local association is measured by the local similarity (LS) score. For time series data of two factors with normalized levels *X*_1_,*X*_2_,⋯,*X*_*n*_ and *Y*_1_,*Y*_2_,⋯,*Y*_*n*_, the LS score is defined as the maximized absolute value of summation $S = \sum _{k = 0}^{l-1} X_{i+k}Y_{j+k}$, where *I*=[*i*,*i*+*l*−1] and *J*=[*j*,*j*+*l*−1] correspond to the intervals maximizing the summation – to be determined by the Smith-Waterman dynamic programming algorithm [12]. By definition, LS score is proportional to the Pearson’s correlation coefficients (PCC) of the aligned parts of the two series. Its statistical significance can be evaluated by a large number of permutations [3, 6] or using the approximation recently proposed by Xia et al. [13].

While local similarity analysis bases its similarity measure on the similarity of the profile or abundance levels of the factors, others suggested that the similarity of increasing, stabilizing or decreasing trends along the time line can also be strong indicators of associations and developed methods based on this alternative measure. Ji and Tan [7] explored this idea by transforming the changing trend of gene expression profiles of *n* consecutive time points into a *n*−1 time point series corresponding to the status of {*d* *e* *c* *r* *e* *a* *s* *e*, *n* *o* *c* *h* *a* *n* *g* *e*, *i* *n* *c* *r* *e* *a* *s* *e*} in expression levels. All possible local associations of a specific length of time span were analyzed by an exhaustive search algorithm to find clusters of genes with significant locally similar expression profiles. Later, He and Zeng [14] renovated the analysis using a dynamic programming algorithm and employed a permutation approach to evaluate the statistical significance for the local trend scores. The techniques used by He and Zeng [14] were similar to those used in local similarity analysis except that the original time series data were first transformed to changing trends series. We will thus refer to the local similarity analysis techniques performed on the transformed changing trends series as the local trend (a.k.a. shape) analysis (LTA) and its corresponding similarity measure as the local trend (LT) score.

Local trend analysis has since been extended and applied to a wide range of biological applications, such as gene-gene association networks [15–17], gene-metabolite networks [18], and transcription factor networks [19–21]. However, one of the major limitations common to local trend analysis is the time consuming permutation procedure used to evaluate the statistical significance (*p*-value) of the LT score. While in practice false discovery rate (FDR or *q*-value) [22] is used to mitigate the multiple comparison problem, still, fast and efficient approximation for the statistical significance of the LT score is urgently needed to estimate the *p*-value. In addition, Madeira et al. [8] first transformed gene expression data into trends for each gene and developed linear time algorithms to find maximal biclusters. Recently, Goncalves and Madeira [9] extended the biclustering algorithms to allow for time delays [8]. These developments are highly significant by considering groups of genes simultaneously instead of gene pairs. However, the statistical issues related to maximal clusters of gene groups are beyond the scope of this study.

Recently, progress has been made to develop efficient statistical significance approximations for local similarity analysis [13, 23]. We notice that by extending the method proposed in Xia et al. [13], it is also possible to obtain *p*-values of local trend scores more efficiently. In this paper we will describe an extension of Xia et al.’s [13] method to local trend analysis, including the mathematical modelling, algorithm implementation and computational validation with simulations and real data applications. In the Methods section, we first formally introduce the concept of local trend analysis and bring in useful results from related works. We then describe the method to model the transformed trend series using the Markov chain theory in both two and three letter alphabet cases. We also propose an approximation formula and numerical computation methods for the statistical significance of LT score based on these models. In the Results and Discussion section, we validate and show the efficiency of our new approach using simulated and real datasets and analyze a real microbial ecological time series dataset from the next generation sequencing (NGS) of marine samples collected near the Polymouth Marine Laboratory (PML) by Gilbert et al. [24].

The major difference between this paper and Xia et al. [13] is the study of statistical significance of local trend score here while Xia et al. [13] studied the statistical significance of local similarity scores based on the original time series data. After transformation of the original time series data to trends, the trend variables are highly dependent even if the original data are independent, making the evaluation of statistical significance of LT score challenging. New approximation results on the tail probabilities of the sums of Markov random variables need to be employed to derive an approximate formula to calculate the statistical significance of LT scores.

## Methods

### The local trend analysis

The first step in local trend analysis is to discretize the factor profile into a changing trend alphabet *Σ* – a set of symbols of interest, which represent distinctive changing trend states [8]. Typically either two letter alphabet *Σ*={*D*,*U*} or simply *Σ*={−1,1} for *trend-down* and *trend-up* states [25, 26], or three letter alphabet *Σ*={*D*,*N*,*U*} or simply *Σ*={−1,0,1} for *trend-down*, *no-change* and *trend-up* states [7, 27] are used. Discretization into larger size alphabet is possible but seldom used in practice. For given time series *X*_1_,*X*_2_,⋯,*X*_*n*_, We transform the *n*-dimensional vector *X* to a *n*−1 dimensional trend vector ${d_{i}^{X}}, i = 1, 2, \cdots, n-1$, by the following rules. When *X*_*i*_≠0,

(1) $$\begin{array}{@{}rcl@{}}  {d_{i}^{X}} =\left\{ \begin{array}{cl} 1 & \text{if}\;\; \frac{X_{i+1}-X_{i}}{|X_{i}|}\geq t \\ 0 & \text{if}\;\; -t<\frac{X_{i+1}-X_{i}}{|X_{i}|}<t \\ -1 & \text{if}\;\; \frac{X_{i+1}-X_{i}}{|X_{i}|}\leq-t \\ \end{array} \right., \end{array} $$

where *t*≥0 is a threshold value for declaring changing trends. When *X*_*i*_=0, ${d_{i}^{X}}$ is defined as:

(2) $$\begin{array}{@{}rcl@{}}  {d_{i}^{X}} =\left\{ \begin{array}{cl} 1 & \;\text{if}\; X_{i} = 0 \;\text{and}\; X_{i+1} > 0 \\ 0 & \;\text{if}\; X_{i} = 0 \;\text{and}\; X_{i+1} = 0 \\ -1 & \;\text{if}\; X_{i} = 0 \;\text{and}\; X_{i+1} < 0 \\ \end{array}\right.. \end{array} $$

**Fig. 1:**
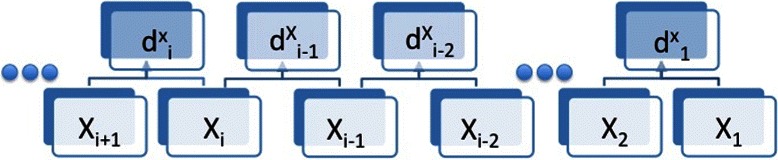
Generation of the trend series. The original series *X* is changed into the trend series *d* ^*X*^ using the discretizing rules given in equations 1 and 2. Note that the value of ${d^{X}_{i}}$ depends solely on the values of *X*_*i*+1_ and *X*_*i*_ but not any other values of X

The trend series generating process and the dependency between *X*_*i*_’s and ${d^{X}_{i}}$’s are depicted in Fig. 1. These rules were formalized in Ji and Tan [7] and Madeira et al. [8].

Based on this data transformation, the subsequent algorithms and statistics of local trend analysis closely follow that for local similarity analysis [3, 5, 6]. That is, for a pair of transformed trend series ${d_{1}^{X}}, {d_{2}^{X}}, \cdots, d_{n-1}^{X}$ and ${d_{1}^{Y}}, {d_{2}^{Y}}, \cdots, d_{n-1}^{Y}$, the Smith-Waterman dynamic programming algorithm [6, 12] is used to find the interval pair *I*=[*i*,*i*+*l*−1] and *J*=[*j*,*j*+*l*−1] of the same length *l* with |*i*−*j*|≤*D* such that the absolute value of $S = \sum _{k = 0}^{l-1} d^{X}_{i+k}d^{Y}_{j+k}$ is maximized, which we refer to as local trend (LT) score with maximum time delay *D*, where *D* is a pre-defined parameter. Statistical significance for LT score corresponds to the probability of observing such a score or larger under the null hypothesis that the two factors *X* and *Y* are not associated. It was used to be approximated by permuting one of the time series data many times and calculating the fraction of times that the LT score for the permuted data is higher than that for the original data [3, 14]. With the permutation approach, the observations for the samples at the different time points are assumed independent under the null model.

### Approximate statistical significance for local trend analysis

The permutation procedures described above to approximate the statistical significance for local trend analysis have several drawbacks. First, the calculated *p*-values have substantial inherent variability associated with the randomness in permutation unless the number of permutations is very large. Second, the procedure is computationally expensive– the computational time scales linearly with the inverse of the required *p*-value precision, which is prohibitive for all-versus-all pairwise analysis of high-throughput datasets.

In fact, the asymptotic theories for the tail distribution of the range of partial sum of zero-mean independent, identically distributed (i.i.d.) and first order Markov chain exist [28–30] and can be applied here to calculate *p*-values under the null model. Formulae for fast and efficient approximation of the statistical significance for aligning two i.i.d. zero-mean sequences had been obtained and successfully applied to local similarity analysis previously [13]. In contrast, in local trend analysis, even if the original series *X*_*i*_’s are considered independent, the transformed trend series ${d_{i}^{X}}, i = 1, 2, \cdots, n-1$ are not independent because, for any consecutive pair ${d_{i}^{X}}$ and $d_{i+1}^{X}$, they both depend on *X*_*i*_ (as shown in Fig. 1). They are not even a Markov chain of any order. In order to use the theory in [28–30] to approximate the statistical significance of LT scores, we make several simplifying assumptions.

The first assumption is that the time series data *X*_*i*_,*i*=1,2,⋯,*n* and *Y*_*i*_,*i*=1,2,⋯,*n* are exchangeable in that any order of the sample is equally likely. Time series data generally do not follow the exchangeability assumption and usually follow some trends. In particular, the value at a particular time may depend on the value at a previous time point. One way to overcome this complexity is to regress the value at time *t*+1 with respect to the value at the previous time point *t* and use the resulting residue for the follow up analysis. In the following of the paper, we assume that such transformations have been carried out and the exchangeability assumption as in most studies in the literature holds.

Secondly, we naively assume the first order Markov chain model for ${d_{i}^{X}}, i = 1, 2, \cdots $. As stated above, this assumption is obviously incorrect. We make this assumption for the convenience of using the theory in [28–30]. We also assume that the product of a pair of independent trend series ${d^{X}_{i}} {d^{Y}_{i}}$ follows a first order Markov chain, i.e.,

(3) $$\begin{array}{*{20}l} &P\left(\left(d^{X}d^{Y}\right)_{i}|\left(d^{X}d^{Y}\right)_{i-1}, \ldots,\ \left(d^{X}d^{Y}\right)_{1}\right)\\ &\qquad \approx \quad \!\! P\left(\left(d^{X}d^{Y}\right)_{i}|\left(d^{X}d^{Y}\right)_{i-1}\right). \end{array} $$

Under the assumption that *X* and *Y* have supports in an interval, ${d^{X}_{i}} {d^{Y}_{i}}$ is irreducible and aperiodic so that the theories for Markov random variables in [28–30] can be adapted.

Thirdly, we make the simplifying assumption that the LT scores for different time delays are independent when we do local trend analysis allowing time delays. Since the LT scores for different delays are all calculated based on the same values of *X* ^′^s and *Y* ^′^s, this independent assumption is violated. We make this assumption purely for computational convenience.

We note the lack of mathematical rigor for approximating the p-value in this study. Therefore, the approaches presented in this paper can only be regarded as heuristic and should not be regarded as rigorous mathematical approximations. We show the usefulness of our approximation by comparing the approximate p-value with that obtained from a large number of permutations. They are close in the sense that the first no-zero decimals of the p-values from the approximation and the permutations are mostly the same. The simulations also show that the approximate p-value is slightly larger than that obtained through permutations. Due to the conservativeness of the approximate p-value, hypothesis testing for associated pairs of factors based on the approximate p-value may have lower power compared to that based on more accurate p-values. We recommend a hybrid approach to combine approximation with permutations to obtain associated pairs of factors without lowering the power. The conservative nature of the approximate p-value allows us to first calculate the approximate p-values for all pairs of factors and then use permutations to obtain the more accurate p-values only for factor pairs with approximate p-value less than a loose threshold. This practice significantly saves computational time as most factor pairs have relatively large approximate p-values. Future studies on more accurate approximation of statistical significance for LT scores based on rigorous mathematical theory are needed.

Using the theory of Bachelier-Wiener processes, Feller [28] studied the approximate distribution of the range *R*_*n*_ of the partial sum of *n* i.i.d. random variables $\{Z_{i}\}_{i=1}^{n}$ with mean 0 and variance *σ*. Daudin et al. [29] studied the distribution of the maximum partial sum of either i.i.d. random variables or an irreducible aperiodic first order Markov chain taken values on a finite subset of the real line. Let *φ* be the stationary distribution of the Markov chain *Z*_*i*_, *i*=1,2,⋯, *E*_*φ*_(*Z*_1_)=0 and

(4) $$\begin{array}{@{}rcl@{}}  \sigma^{2} = E_{\varphi}\left({Z_{1}^{2}}\right) + 2 \sum\limits_{k = 1}^{\infty} E_{\varphi}\left(Z_{1} Z_{k+1}\right). \end{array} $$

Based on these results, it can be shown

(5) $$ {\fontsize{9}{6}\begin{aligned} \mathcal{L}(x) =& {\lim}_{n \rightarrow \infty} P\left \{\frac{R_{n}}{\sqrt{n} \sigma} \geq x \right \} \\ =& 1 - 8 \sum\limits_{k = 1}^{\infty} \left (\frac{1}{x^{2}} + \frac{1}{(2k-1)^{2} \pi^{2}} \right)\exp \left(- \frac{(2k-1)^{2} \pi^{2}}{2x^{2}} \right),  \end{aligned}}  $$

where *R*_*n*_ is the range of partial sums of *Z*_1_,*Z*_2_,⋯,*Z*_*n*_. We will use this equation to approximate the statistical significance of local trend score. For local trend analysis with no time delays, we let $Z_{i} = {d_{i}^{X}} {d_{i}^{Y}}$ and approximate *Z*_*i*_ by a first order Markov chain. Then the statistical significance of LT score without time delays (*D*=0) can be approximated using equation (5). With time delay of at most *D*, using a similar argument as in [13] and assuming that the LT scores for different delays are independent, we can approximate the statistical significance (p-value) of a LT score with delay at most *D* by

(6) $$ \begin{array}{ll} \mathcal{L}_{D}(x) =& P\left(LS(D)/(\sigma \sqrt{n}) \geq x\right)\\ & \approx 1 - 8^{2D + 1} \left(\sum\limits_{k = 1}^{\infty} \left(\frac{1}{x^{2}} + \frac{1}{\left(2k-1\right)^{2}\pi^{2}} \right)\right.\\ &\left.\exp \left(- \frac{\left(2k-1\right)^{2} \pi^{2}}{2x^{2}} \right) {\vphantom{\sum\limits_{k = 1}^{\infty}}}\right)^{2D + 1}. \end{array}  $$

### The Markov chain model: two letter alphabet case

We first propose a Markov chain model for local trend analysis with the relatively simple two letter alphabet case (i.e. *t*=0), for which an exact solution for *σ* is available. Consider *X*_1_,*X*_2_,⋯,*X*_*n*_ as continuous random variables such that the probability of taking a fixed value to be 0. By order statistics, we have $P\left [({d_{i}^{X}}, d_{i+1}^{X}) = (1,1)\right ] = P[({d_{i}^{X}}, d_{i+1}^{X}) = (-1,-1)] = 1/6$ and $P\left [\left ({d_{i}^{X}}, d_{i+1}^{X}\right) = \left (1,-1\right)\right ] = P\left [\left ({d_{i}^{X}}, d_{i+1}^{X}\right)=\left (-1,1\right)\right ] = 1/3$ and $P({d_{i}^{X}} = 1) = P({d_{i}^{X}} = -1) = 1/2$ if the *X*_*i*_’s are exchangeable. Assuming that ${d_{i}^{X}}$’s form a first order Markov chain, we can solve for the transition matrix



Then it can be shown by spectral expansion [31] that

$$T^{k} = \frac{1}{2} \left(\begin{array}{ll} 1 + (-1)^{k}/3^{k} & 1 - (-1)^{k}/3^{k}\\ 1 - (-1)^{k}/3^{k} & 1 + (-1)^{k}/3^{k} \end{array} \right). $$

For *k*≥1, we have $P\left ({d_{1}^{X}} d_{k+1}^{X} = 1\right) = \left (1 + (-1)^{k}/3^{k}\right)/2$ and $P\left ({d_{1}^{X}} d_{k+1}^{X} = -1\right) = \left (1 - (-1)^{k}/3^{k}\right)/2$. Thus, $E\left ({d_{1}^{X}} d_{k+1}^{X}\right) = \left (-1\right)^{k}/3^{k}$. In local trend analysis, we compare ${d_{1}^{X}}, {d_{2}^{X}}, \cdots, d_{n-1}^{X}$ with ${d_{1}^{Y}}, {d_{2}^{Y}}, \cdots, d_{n-1}^{Y}$. Therefore, we have $\sigma _{d^{X}d^{Y}}^{2} = E\left (\left ({d_{1}^{X}}\right)^{2}\right) E\left (\left ({d_{1}^{Y}}\right)^{2}\right)+ 2 \sum _{k = 1}^{\infty } E\left ({d_{1}^{X}} d_{k+1}^{X}\right) E\left ({d_{1}^{Y}} d_{k+1}^{Y}\right) = 1 + 2 \sum _{k = 1}^{\infty } 1/3^{2k} = 1 + 1/4 = 1.25$. When *D*=0, LT score for local trend analysis is the range of partial sum $\sum _{i}{d_{i}^{X}} {d_{i}^{Y}}$. Following the result presented in equation (4), we obtain the approximate formula for local trend score *p*-value in the two letter alphabet case (i.e. *t*=0):

(7) $$\begin{array}{*{20}l}  P(LT(D) \geq s_{D}) &= P\left(\frac{LT(D)}{\sigma_{d^{X}d^{Y}}\sqrt{n}} \geq \frac{s_{D}}{\sigma_{d^{X}d^{Y}}\sqrt{n}} \right)\\ &= \mathcal{L}_{D} \left(\frac{s_{D}}{\sqrt{1.25\times n}} \right), \end{array} $$

where the function ${\mathcal L}_{D}$ is defined in equation (6) and *s*_*D*_ is the LT score with delay at most *D*.

### The Markov chain model: the three letter alphabet case

We next show the Markov chain modeling for local trend analysis with the three letter alphabet case (i.e. *t*>0) – allowing a more flexible description of state changes. In this case, the transition matrix *T*(*t*) is a function of the threshold value *t*. However, a closed form formula for *T*(*t*) is not readily available for general zero-mean i.i.d. random variable *X*_*i*_’s. Instead, we have to use Monte Carlo strategy to numerically approximate *T*(*t*) for a given threshold value *t*.

To do the Monte Carlo simulation, we first generate a series of i.i.d. standard normal random values *X*_1_,…,*X*_*N*_ for *N* large and use rules in equations (1) and (2) to transform the series into trend series ${d_{1}^{X}}, \ldots, d_{N-1}^{X}$. We approximate the transition probability from *a* to *b* by *T*(*t*)_*a*,*b*_=*C*_*a*,*b*_/*C*_*a*_, *a*,*b*=−1,0,1, where *C*_*a*,*b*_ is the number of pairs such that $\left ({d^{X}_{i}}, d^{X}_{i+1}\right) = (a, b), ~i = 1, 2, \cdots, N-1$ and *C*_*a*_ is the number of pairs such that ${d^{X}_{i}} = a$. In this study, we let *N*=10000. Because all the rows of *T*(*t*) sum to 1, using the symmetry condition, we have *T*(*t*)_1,1_=*T*(*t*)_−1,−1_=*b*, *T*(*t*)_1,−1_=*T*(*t*)_−1,1_=*c*, *T*(*t*)_0,1_=*T*(*t*)_0,−1_=*d*, *T*(*t*)_1,0_=*T*(*t*)_−1,0_=1−*b*−*c* and *T*(*t*)_0,0_=1−2*d* and therefore *T*(*t*) is of the following form:



Any row of the infinity power of *T*(*t*), *T* ^*∞*^(*t*), converge to the stationary distribution *φ*. So we only need to estimate *b*,*c*,*d* to obtain *T*(*t*) and *φ*, reducing the number of parameters to be estimated to three. Though numerical, this Monte Carlo approach is very fast and accurate given today’s computational power.

With *T*(*t*) known, its eigenvalues $\{\lambda _{i}(t)\}_{i=1}^{3}$, right column eigenvectors $\{r_{i}(t)\}_{i=1}^{3}$ and left column eigenvectors $\{l_{i}(t)\}_{i=1}^{3}$ are readily solvable. To be concise, we simply omit the dependence on *t* in notation and denote *λ*(*t*), *r*(*t*), *l*(*t*) and *T*(*t*) in shorthand by *λ*, *r*, *l* and *T*. The property of transition matrix of aperiodic and irreducible Markov chain guarantees *λ*_1_=1, *r*_1_=1 (a three dimensional vector of all 1s) and *φ*=*l*_1_. Using spectral expansion, we can expand the *k*-th power of *T*, *T* ^*k*^, as:

$$\begin{array}{@{}rcl@{}} T^{k} = \sum\limits_{i=1}^{3}{\lambda_{i}^{k}} r_{i}l_{i}' = {\bf1}l_{1}' + \sum\limits_{i=2}^{3}{\lambda_{i}^{k}} r_{i}l_{i}', \end{array} $$

where the individual entry $T^{k}_{u,v}=\sum _{i=1}^{3}{\lambda _{i}^{k}} r_{i,u}l_{i,v}$. Actually carrying out the expansion, we obtain:

(8) $$\begin{array}{@{}rcl@{}} T^{k} = \left(\begin{array}{ccc} \frac{1}{2}(b-c)^k+\frac{(-1+b+c) (b+c-2 d)^{k}}{2 (-1+b+c-2 d)}-\frac{d}{-1+b+c-2 d} & \ \ \ \ \frac{-1+b+c}{-1+b+c-2 d}+\frac{(1-b-c) (b+c-2 d)^{k}}{-1+b+c-2 d} & \ \ \ \ -\frac{1}{2} (b-c)^k+\frac{(-1+b+c) (b+c-2 d)^{k}}{2 (-1+b+c-2 d)}-\frac{d}{-1+b+c-2 d} \\ \quad -\frac{d}{-1+b+c-2 d}+\frac{(b+c-2 d)^{k} d}{-1+b+c-2 d} &\quad \frac{-1+b+c}{-1+b+c-2 d}+\frac{2 (1-b-c) (b+c-2 d)^{k} d}{(-1+b+c) (-1+b+c-2 d)} & \quad\quad -\frac{d}{-1+b+c-2 d}+\frac{(b+c-2 d)^{k} d}{-1+b+c-2 d} \\ -\frac{1}{2} (b-c)^k+\frac{(-1+b+c) (b+c-2 d)^{k}}{2 (-1+b+c-2 d)}-\frac{d}{-1+b+c-2 d} & \ \ \ \ \frac{-1+b+c}{-1+b+c-2 d}+\frac{(1-b-c) (b+c-2 d)^{k}}{-1+b+c-2 d} & \frac{1}{2} (b-c)^k+\frac{(-1+b+c) (b+c-2 d)^{k}}{2 (-1+b+c-2 d)}-\frac{d}{-1+b+c-2 d} \end{array} \right)  \end{array} $$

and let *k*→*∞*, we have the stationary distribution:

(9) $$ \varphi =\left(\begin{array}{lll} \frac{d}{1 - b - c + 2d}, & \frac{1-b-c}{1-b-c+2 d}, & \frac{d}{1 - b - c + 2d} \end{array} \right).  $$

Subsequently, we have

$$\begin{array}{@{}rcl@{}} P\left({d_{1}^{X}} d_{k+1}^{X} = 1\right) & = & P\left(d_{k+1}^{X}=1| {d_{1}^{X}} = 1\right)P\left({d_{1}^{X}}=1\right) \\&&\!\!\!\!\!+ P\left(d_{k+1}^{X}=-1| {d_{1}^{X}} = -1\right)P\left({d_{1}^{X}}=-1\right)  \\ & = & \varphi_{1}T^{k}_{1,1}+\varphi_{-1}T^{k}_{-1,-1}.  \end{array} $$

Similarly,

$$\begin{array}{@{}rcl@{}} P({d_{1}^{X}} d_{k+1}^{X} = -1) & = & \varphi_{1}T^{k}_{1,-1}+\varphi_{-1}T^{k}_{-1,1}.  \end{array} $$

The symmetry of states 1 and -1 ensures *φ*_1_=*φ*_−1_ in the stationary distribution. Thus, using equation (4) we can compute $\sigma _{d^{X}d^{Y}}(t)\phantom {\dot {i}\!}$ as following:

(10) $$\begin{array}{@{}rcl@{}} \sigma_{d^{X}d^{Y}}^{2}(t) & = &\!E\left(\left({d_{1}^{X}}\right)^{2}\right) E\left(\left({d_{1}^{Y}}\right)^{2}\right)+ 2\sum\limits_{k = 1}^{\infty} E\left({d_{1}^{X}} d_{k+1}^{X}\right)\\ &&\qquad E\left({d_{1}^{Y}} d_{k+1}^{Y}\right)\  \\ & = & \!\left(\varphi_{1}+\varphi_{3}\right)^{2} + 2 \sum\limits_{k = 1}^{\infty} \left(P\left({d_{1}^{X}} d_{k+1}^{X} = 1\right)\right.\\ &&\qquad\left.-P\left({d_{1}^{X}} d_{k+1}^{X} = -1\right)\right)^{2}  \\ & = & \!(\varphi_{1}+\varphi_{3})^{2} + 2 \sum\limits_{k = 1}^{\infty} \left(\varphi_{1}T^{k}_{1,1}+\varphi_{3}T^{k}_{3,3}\right.\\ &&\qquad\left.-\varphi_{1}T^{k}_{1,3}-\varphi_{3}T^{k}_{3,1}\right)^{2}  \\ & = & \!4{\varphi_{1}^{2}} + 2{\varphi_{1}^{2}} \sum\limits_{k = 1}^{\infty} \left(T^{k}_{1,1}-T^{k}_{1,3}+T^{k}_{3,3}-T^{k}_{3,1}\right)^{2}  \\ & = & \!4{\varphi_{1}^{2}} \left(1+ 2\sum\limits_{k=1}^{\infty}\left(b-c\right)^{2k} \right)  \\ & = & \!4\left(\frac{d}{\!1-b-c+2 d}\!\right)^{2}\!\left(\!1\,+\,\frac{2(b-c)^{2}}{1-(b-c)^{2}\!} \right).  \end{array} $$

Since equation (10) can be numerically calculated based on the Monte Carlo estimates of *b*,*c*,*d*, we can calculate $\sigma _{d^{X}d^{Y}}(t)\phantom {\dot {i}\!}$ and then plug it into ${\mathcal L}_{D}$ as defined in equation (6) to obtain:

(11) $$\begin{array}{@{}rcl@{}} P(LT(D) \geq s_{D})& =& P\left(\frac{LT(D)}{ \sigma_{d^{X}d^{Y}}(t) \sqrt{n}} \geq \frac{s_{D}}{\sigma_{d^{X}d^{Y}}(t)\sqrt{n}} \right) \\&=& \mathcal{L}_{D} \left(\frac{s_{D}}{\sigma_{d^{X}d^{Y}}(t)\sqrt{n}} \right),  \end{array} $$

which is the final formula for approximating the *p*-values of LT scores in the three letter alphabet case.

We compare the approximate p-values calculated using equation (6) and the p-value using simulations. We then apply our method to analyze three real datasets. The first one is a microarray gene expression dataset of yeast cell division cycles (referred to as ‘CDC’), synchronized by the cdc-15 gene from Spellman et al. [32]. The second one is a human microbiota dataset from one male (M3) and one female (F4) sampled daily at three body sites (feces, mouth and palms) for 15 months (M3) and for 6 months (F4) from the motion picture of human microbiome paper by Caporaso et al. (referred to as ‘MPH’) [33]. The third one is a microbial ecological time series data from recent NGS of marine microbial community samples collected from sites close to the Polymouth Marine Laboratory (PML) [24] (referred to as ‘PML’). We apply local trend analysis (with *t*=0 and *t*=0.5) to analyze the first two datasets and compared the approximate and permutation *p*-values. We are the first to analyze the third dataset using local trend analysis and found interesting results.

## Results and Discussion

### Simulation Studies

#### Monte Carlo estimates of the transition probabilities

In deriving the approximate statistical significance, i.e. *p*-values, for local trend analysis, we make simplifying assumptions to use Markov chain modeling on ${d_{i}^{X}}$ and ${d_{i}^{Y}}$. However, the validity and accuracy of the approximations have to be evaluated. Thus, we first study whether the transition probabilities estimated based on simulated time series data are close to those approximated using the Markov chain theory. We demonstrate this when *X*_*i*_’s and *Y* *i*′*s* are i.i.d. standard normal random variables, because in most common applications, raw biological experimental series data are normalized before pairwise comparisons. We use 10,000 Monte Carlo randomly generated *X*_*i*_ and *Y*_*i*_’s, transform them with the thresholds *t*=0 and *t*=0.5 and estimate the parameters *b*, *c*, and *d* in the probability transition matrix. Meanwhile the transition matrix of the Markov chain is still solvable by integration using the Mathematica software.

In Table 1, for all the thresholds studied, the numerical integration results are very close to that learned from the randomly generated series. For example, when *t*=0, the estimated parameters are (*b*=0.3342,*c*=0.6658) while the Markov chain theory yields (*b*=0.3333,*c*=0.6667). When *t*=0.5, the estimates are (*b*=0.2313,*c*=0.6083,*d*=0.4034) and the Markov chain theory numerical results are (*b*=0.2311,*c*=0.6088,*d*=0.4043). With 10,000 simulations, the estimates differ at the third or fourth decimals and the Monte Carlo calculation is done within only seconds. With a larger number of simulations, the precision can be even better. Therefore our Markov chain modeling approximates the state transition probabilities and stationary distribution efficiently and accurately.

**Table 1:** The estimated parameters of the probability transition matrix using Monte Carlo simulations are very close to that based on numerical integration using Mathematica for all cases studied: *t*={0,0.5,1,2}. Parameters *b*,*c*,*d* as introduced in equation (8) are sufficient parameters to describe such a Markov chain. P. = parameters, Num. Int. = numerical integration, N.A. = not applicable

	P.	Monte Carlo	Num. Int.
t=0	b	0.3342	0.3333
	c	0.6658	0.6667
	d	N.A.	N.A.
t=0.5	b	0.2313	0.2311
	c	0.6083	0.6088
	d	0.4034	0.4043
t=1	b	0.1265	0.1268
	c	0.4998	0.5000
	d	0.3432	0.3429
t=2	b	0.0310	0.0303
	c	0.1608	0.1617
	d	0.2203	0.2199

#### Approximating the tail probability of the LT score using equation (6)

The approximate *p*-value for the local trend score given in the Methods section is only applicable when the *p*-value is small and the number of time points is large. Therefore, we first study the range of applicability of our approximation formulae. For the two alphabet case, we pre-calculate $\sigma _{d^{X}d^{Y}}=\sqrt {1.25}$ for *t*=0. Table 2 gives the approximate tail probability (*p*-value) based on equation (7) (2nd column) and the simulated probability $P(LT(0)/\sqrt {1.25n} \geq x)$ (3rd to 9th columns) for different numbers of time points when *D*=0. It can be seen that the approximate tail probability is close to the simulated probability when the approximate *p*-value is less than 0.05 starting from *n*=20 time points in the sense the first no-zero decimal of the approximate p-value is mostly the same as that of the simulated p-value. In general, the approximate tail probability is slightly larger than the simulated values when *D*=0 (see Table 2). Similar results were observed for *D*=1,2,3 (see Tables 3, 4 and 5). Thus, it will be slightly conservative in declaring significant associations if we use the approximate tail distribution to calculate the *p*-value. However, for relatively small value of *x*, the approximate tail probability can be much larger than the simulated tail probability. Since we are mostly interested in significant associations with small p-values, we do not consider this as a problem. On the other hand, since the approximate p-value is larger than the true p-value, the test based on the approximate p-value is conservative and the power of the test can be lower than the power based on the true p-value, which can be approximated by simulations.

**Table 2:** Approximation for the tail probability of local trend score (LT score) versus the simulated probability $P(LT(D)/\sqrt {1.25 n} \geq x)$. The approximate probability based on equation (7) is given in the 2nd column and the probability that $LT(D)/\sqrt {1.25n} \geq x$ from simulations is given in the 3rd to the 9th columns. Here, *D*=0

		The number of time points *n*						
x	Approximation	10	20	30	40	60	80	100
2.0	0.1815	0.0483	0.1284	0.0948	0.0974	0.1148	0.1405	0.1304
2.2	0.1111	0.0483	0.0717	0.0595	0.0663	0.0613	0.0853	0.0799
2.4	0.0656	0.0121	0.0419	0.0358	0.0455	0.0428	0.0481	0.0465
2.6	0.0373	0.0042	0.0205	0.0217	0.0174	0.0222	0.0283	0.0223
2.8	0.0204	0.0042	0.0111	0.0071	0.0103	0.0107	0.0157	0.0127
3.0	0.0108	0.0000	0.0048	0.0035	0.0038	0.0077	0.0076	0.0070
3.2	0.0055	0.0000	0.0021	0.0017	0.0014	0.0034	0.0037	0.0036
3.4	0.0027	0.0000	0.0001	0.0008	0.0003	0.0021	0.0013	0.0016
3.6	0.0013	0.0000	0.0001	0.0002	0.0003	0.0007	0.0011	0.0003
3.8	0.0006	0.0000	0.0001	0.0001	0.0001	0.0004	0.0006	0.0002
4.0	0.0003	0.0000	0.0000	0.0000	0.0000	0.0001	0.0003	0.0002
4.2	0.0001	0.0000	0.0000	0.0000	0.0000	0.0000	0.0002	0.0000

**Table 3:** Approximation for the tail probability of local trend score (LT score) versus the simulated probability $P(LT(D)/\sqrt {1.25 n} \geq x)$. The approximate probability based on equation (7) is given in the 2nd column and the probability that $LT(D)/\sqrt {1.25 n} \geq x$ from simulations is given in the 3rd to the 9th columns. Here, *D*=1

		The number of time points *n*						
x	Approximation	10	20	30	40	60	80	100
2.0	0.4516	0.0840	0.2674	0.2184	0.2350	0.2711	0.3295	0.3054
2.2	0.2977	0.0840	0.1576	0.1422	0.1659	0.1483	0.2034	0.2007
2.4	0.1841	0.0285	0.0855	0.0899	0.1118	0.1099	0.1206	0.1236
2.6	0.1077	0.0041	0.0447	0.0527	0.0478	0.0582	0.0663	0.0541
2.8	0.0601	0.0041	0.0192	0.0150	0.0291	0.0271	0.0335	0.0326
3.0	0.0320	0.0000	0.0078	0.0069	0.0092	0.0188	0.0173	0.0189
3.2	0.0164	0.0000	0.0028	0.0037	0.0065	0.0080	0.0081	0.0098
3.4	0.0081	0.0000	0.0004	0.0015	0.0025	0.0035	0.0027	0.0030
3.6	0.0038	0.0000	0.0004	0.0002	0.0017	0.0017	0.0022	0.0015
3.8	0.0017	0.0000	0.0002	0.0000	0.0007	0.0011	0.0007	0.0007
4.0	0.0008	0.0000	0.0000	0.0000	0.0000	0.0002	0.0003	0.0002
4.2	0.0003	0.0000	0.0000	0.0000	0.0000	0.0001	0.0001	0.0001
4.4	0.0001	0.0000	0.0000	0.0000	0.0000	0.0000	0.0000	0.0000
4.6	0.0001	0.0000	0.0000	0.0000	0.0000	0.0000	0.0000	0.0000

**Table 4:** Approximation for the tail probability of local trend score (LT score) versus the simulated probability $P(LT(D)/\sqrt {1.25 n} \geq x)$. The approximate probability based on equation (7) is given in the 2nd column and the probability that $LT(D)/\sqrt {1.25 n} \geq x$ from simulations is given in the 3rd to the 9th columns. Here, *D*=2

		The number of time points *n*						
x	Approximation	10	20	30	40	60	80	100
2.0	0.6326	0.1148	0.3733	0.3108	0.3358	0.3966	0.4744	0.4284
2.2	0.4452	0.1148	0.2235	0.2013	0.2380	0.2355	0.3145	0.2864
2.4	0.2876	0.0306	0.1227	0.1258	0.1633	0.1749	0.1884	0.1825
2.6	0.1730	0.0057	0.0575	0.0768	0.0682	0.0908	0.1064	0.0829
2.8	0.0981	0.0057	0.0267	0.0219	0.0441	0.0419	0.0571	0.0480
3.0	0.0528	0.0000	0.0109	0.0110	0.0153	0.0287	0.0274	0.0250
3.2	0.0272	0.0000	0.0037	0.0044	0.0081	0.0120	0.0128	0.0119
3.4	0.0134	0.0000	0.0005	0.0018	0.0018	0.0045	0.0051	0.0037
3.6	0.0063	0.0000	0.0005	0.0004	0.0009	0.0015	0.0035	0.0018
3.8	0.0029	0.0000	0.0000	0.0001	0.0003	0.0009	0.0014	0.0008
4.0	0.0013	0.0000	0.0000	0.0000	0.0001	0.0002	0.0005	0.0002
4.2	0.0005	0.0000	0.0000	0.0000	0.0001	0.0001	0.0002	0.0000
4.4	0.0002	0.0000	0.0000	0.0000	0.0000	0.0000	0.0000	0.0000
4.6	0.0001	0.0000	0.0000	0.0000	0.0000	0.0000	0.0000	0.0000

**Table 5:** Approximation for the tail probability of local trend score (LT score) versus the simulated probability $P(LT(D)/\sqrt {1.25 n} \geq x)$. The approximate probability based on equation (7) is given in the 2nd column and the probability that $LT(D)/\sqrt {1.25 n} \geq x$ from simulations is given in the 3rd to the 9th columns. Here, *D*=3

		The number of time points *n*						
x	Approximation	10	20	30	40	60	80	100
2.0	0.7539	0.1130	0.4402	0.3841	0.4214	0.4882	0.5808	0.5319
2.2	0.5616	0.1130	0.2688	0.2530	0.3015	0.2901	0.3974	0.3751
2.4	0.3779	0.0278	0.1451	0.1643	0.2079	0.2164	0.2475	0.2473
2.6	0.2336	0.0047	0.0762	0.0992	0.0869	0.1131	0.1471	0.1165
2.8	0.1346	0.0047	0.0324	0.0308	0.0535	0.0538	0.0804	0.0682
3.0	0.0732	0.0000	0.0130	0.0169	0.0171	0.0344	0.0423	0.0393
3.2	0.0379	0.0000	0.0048	0.0072	0.0084	0.0140	0.0198	0.0206
3.4	0.0187	0.0000	0.0005	0.0041	0.0024	0.0046	0.0061	0.0060
3.6	0.0089	0.0000	0.0005	0.0008	0.0010	0.0016	0.0037	0.0026
3.8	0.0040	0.0000	0.0000	0.0003	0.0005	0.0008	0.0015	0.0015
4.0	0.0018	0.0000	0.0000	0.0002	0.0001	0.0002	0.0003	0.0009
4.2	0.0007	0.0000	0.0000	0.0000	0.0000	0.0001	0.0001	0.0002
4.4	0.0003	0.0000	0.0000	0.0000	0.0000	0.0000	0.0000	0.0000
4.6	0.0001	0.0000	0.0000	0.0000	0.0000	0.0000	0.0000	0.0000

In many studies, investigators calculate the *p*-values by permuting the time series many times. Next, we compare the permutational and approximate *p*-values by simulations. For the simulated data in the last paragraph, we calculate the *p*-values using both the permutation approach (*P*_*perm*_) and the approximate formulae (*P*_*theo*_) for exactly the same pair of time series data. We do 1000 permutations for each pair and the maximum resolution (precision) of *P*_*perm*_ is 0.001.

**Fig. 2:**
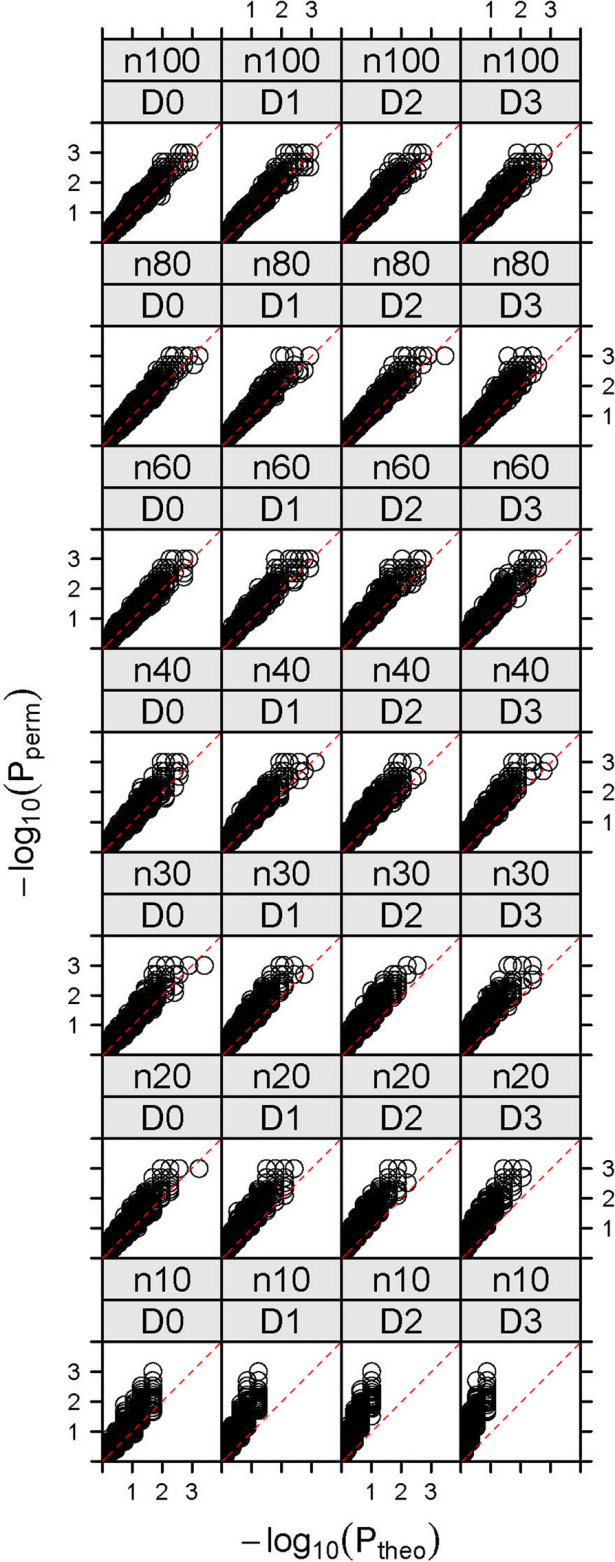
Local trend analysis (*t*=0). The values of *P*_*theo*_ vs *P*_*perm*_ for 10,000 pairs of simulated data. Columns D0 to D3 are for *D*=0,1,2,3. Rows n10 to n100 are for *n*=10,20,30,40,60,80,100

Figure 2 shows the comparison between (*P*_*perm*_) and (*P*_*theo*_). We find at *D*=0, starting from *n*=20 to 30, points in scatter plots become concentrated on the diagonal line (where *P*_*perm*_= *P*_*theo*_) and they become more aligned to the diagonal as *n* increases. This indicates an increasing rate of agreement between the approximate and permutation *p*-values as a good approximation. The same is true with *D*=1,2,3 as the approximation become significantly closer to the permutation results when *n* increases and starting from *n*=30 to 40. However, as the time delay increases, the approximation becomes increasingly less accurate. In summary, we see that if we are interested in statistical significance at a given type I error threshold, the approximation provides results comparable to that from permutations starting from *n*=30 although the theoretical p-value is slightly conservative.

The simulation results for *t*=0.5 are presented in Additional file 1.

### The CDC dataset

**Fig. 3:**
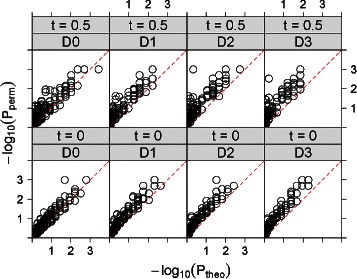
The values of *P*_*theo*_ and *P*_*perm*_ for all-to-all pairwise local trend analysis (*t*=0 and *t*=0.5) of 25 gene expression profiles from the CDC dataset. Rows are for *t*=0,0.5, respectively. Columns D0 to D3 are for *D*=0,1,2,3, respectively

The CDC dataset consists of the expression profiles of 6,177 genes at 24 time points. It is extremely time consuming to approximate the *p*-values for local trend analysis for all the gene pairs using permutations. Thus, we only randomly select 25 genes and estimate the *p*-value for each of the 300 gene pairs by permuting the original data 1000 times. We then compare *P*_*theo*_ from our approximation to *P*_*perm*_ from the permutation approach, as shown in Fig. 3. For both *t*=0 and *t*=0.5 and *D*=0,1,2,3, it can be seen from the figure that *P*_*theo*_ is highly positively correlated with *P*_*perm*_, but *P*_*theo*_ is slightly higher than *P*_*perm*_ indicating that it is conservative when we declare statistical significance using *P*_*theo*_.

For all the situations considered, among the gene pairs with *P*_*perm*_≤0.05, over half of them are declared as significant by *P*_*theo*_. For the *t*=0 case, none of *P*_*theo*_ is less than 0.05 when *P*_*perm*_>0.05. With *D*=0, we have 29 (10 %) out of 300 found significant while 260 (87 %) non-significant by both approaches, and in total 289 (97 %) are in agreement. Among the gene pairs with *P*_*perm*_>0.05, none of them are significant using *P*_*theo*_. Among the gene pairs declared as significant by *P*_*perm*_, about 29/40 (73 %) are declared as significant by *P*_*theo*_. Similarly, with *D*=1,2,3, there are 286 (95 %), 284 (95 %) and 285 (95 %) *p*-value pairs in agreement with both *P*_*perm*_ and *P*_*theo*_, respectively.

For *t*=0.5, with *D*=0, we have 260 (87 %) out of 300 found to be non-significant by both approximation and permutations. Among the remaining, 28(9 %) are found significant by both methods, and in total 288 (96 %) are in agreement. The results are similar with *D*=1,2,3, with 284 (95 %), 286 (95 %) and 278 (93 %) in agreement, respectively. Moreover, all-to-all pairwise analysis of the whole CDC dataset with *D*=3 and permutation 1000 times cannot be completed in 100 hours on a “Dell, PE1950, Xeon E5420, 2.5GHz, 12010MB RAM” computing node, while, using the approximate approach, it can be finished within two hours on the same computing node.

### The MPH dataset

**Fig. 4:**
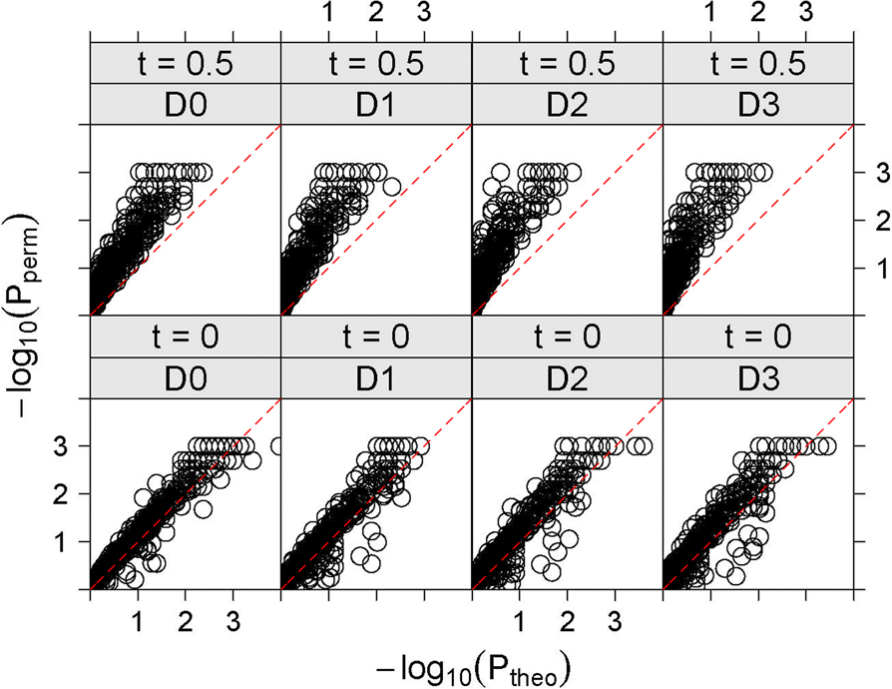
The values of *P*_*theo*_ and *P*_*perm*_ for all-to-all pairwise local trend analysis (*t*=0 and *t*=0.5) of 40 abundant OTUs from the MPH dataset. Rows are for *t*=0,0.5, respectively. Columns D0 to D3 are for *D*=0,1,2,3, respectively

The MPH dataset was collected from two healthy subjects, one male (M3) and one female (F4), both were sampled daily at three body sites (gut (feces), mouth, and skin (left and right palms)) 130, 133 and 135 days, respectively [33]. There are 335, 1295 and 373 unique operational taxonomic units (OTU) from feces, palm and tongue sites of ‘F4’ and ‘M3’, respectively. In order to feasibly finish computational time of the permutation approach, we select 40 abundant OTUs from the right palm of ‘F4’ of the MPH dataset. We present approximate and permutation *p*-value comparison for local trend analysis in Fig. 4. The figure shows that the approximate p-value is close to that from the permutations when *t*=0. However, the approximate p-values are generally much larger than that based on permutations. One potential explanation is the sparsity of the data due to the large number of OTUs.

We choose type-I error threshold to be 0.05. For *t*=0, the results show good agreement. With *D*=0, we have 482 (62 %) and 263 (34 %) out of 780 found non-significant and significant, respectively, by both methods. In total 745 (96 %) are in agreement. Among the 33 (4 %) OTU pairs with discordant significance by *P*_*theo*_ and *P*_*perm*_, all of them are significant by *P*_*perm*_ but non-significant by *P*_*theo*_, which is more conservative. The results are similar with *D*=1,2,3, with 744 (95 %), 743 (95 %) and 732 (94 %) in concordance, respectively, and about 3–4 % incidences significant by *P*_*perm*_ but non-significant by *P*_*theo*_.

For *t*=0.5 and *D*=0, we have 489 (63 %) out of 780 found non-significant and 188 (24 %) significant by both methods. In total, 677 (87 %) are in agreement. All of the discordant 103 (13 %) pairs are significant by *P*_*perm*_ but non-significant by *P*_*theo*_. The results are similar with *D*=1,2,3, where 676 (88 %), 676 (88 %) and 685 (88 %) are in concordance, respectively. There are about 12–13 % associations significant by *P*_*perm*_ but non-significant by *P*_*theo*_, showing that *P*_*theo*_ is more conservative.

### The PML dataset

Gilbert et al. [24] studied the microbial community composition change using high-resolution 16S rRNA tag NGS sequencing of samples taken monthly over 6 years at a temperate marine coastal site off Plymouth Marine Laboratory (PML), Plymouth, UK (total 72 time points). They identified a total of 8,794 different bacterioplankton OTUs and environmental factors, and their presence are most common, abundant and variable across all the samples. As a proof-of-concept analysis, we select 73 abundant OTUs, including 15 environment factors. The taxonomic level to which the OTUs could be identified was Phylum and Class. The raw read counts data were first normalized by percentile and Z-score transformation and then converted into trend series of {−1,0,1} with *t*=0. We then apply the local trend analysis to the trend series and analyze the results below.

**Fig. 5:**
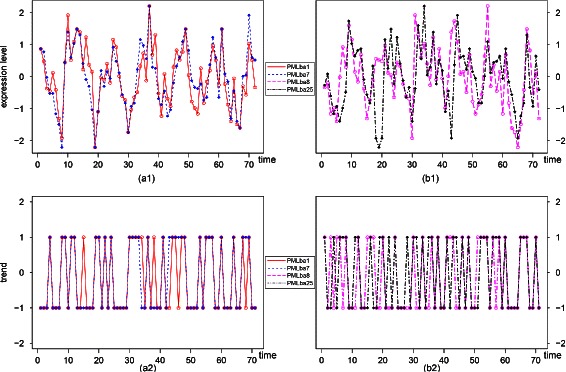
(**a1**): Normalized abundance level series of PMLba1 (Alphaproteobacteria031) and PMLba7 (Alphaproteobacteria032) having the highest positive LT score starting from the 1st time point. (**a2**): Trend series of PMLba1 (Alphaproteobacteria031) and PMLba7 (Alphaproteobacteria032) with a LT score 0.830986 (*D*=0 with *P*=0, *Q*=0.000007 in approximation) starting from the 1st time point. (**b1**): Normalized abundance levels of PMLba8 (Gammaproteobacteria0341) and PMLba25 (Bacteroidetes0326) associated starting from the 8th time point. (**b2**): Trend series of PMLba8 (Gammaproteobacteria0341) and PMLba25 (Bacteroidetes0326) with a LT score 0.56338 (*D*=0 with *P*=0.000598, *Q*=0.022149 in approximation) starting from the 8th time point

In total 77 (81.9 %) of 94 associated OTU pairs (*P*<0.05, *Q*<0.05) are bacteria to bacteria. In those 77 OTU pairs, there are 54 OTU pairs belonging to Proteobacteria, which includes: Alphaproteobacteria, Betaproteobacteria and Gamaproteobacteria; nine pairs of OTUs belong to Bacteroidetes, Cyanobacteria and Verrucomicrobia. While the remaining 14 pairs of OTUs show the inter-group association between Proteobacteria and other bacteria. For example: PMLba1 (Alphaproteobacteria031) and PMLba7 (Alphaproteobacteria032) have the highest positive LT score from time point 1 (*L* *T*=0.830986, *D*=0 with *P*<1*e* ^−16^, *Q*=0.000007). Their abundance level time series and trend series are shown in Fig. 5(a1) and Fig. 5(a2), respectively. PMLba8 (Gammaproteobacteria0341) and PMLba25 (Bacteroidetes0326) with a LT score 0.56338 (*D*=0 with *P*=0.000598, *Q*=0.022149 in approximation), whose abundance level time series and trend series are shown in Fig. 5(b1) and Fig. 5(b2), respectively, have similar trends from the 8th time point and onward.

**Fig. 6:**
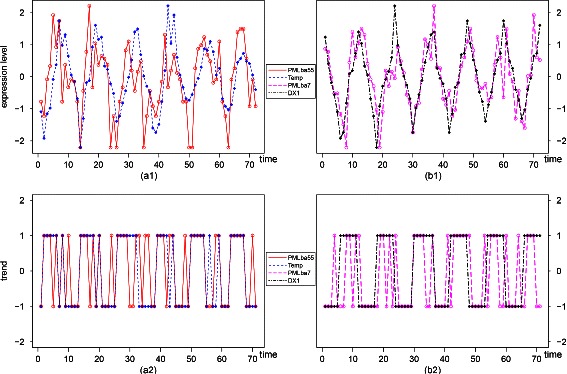
(**a1**): Normalized abundance level series of PMLba55 (Gammaproteobacteria03170) and temperature are associated from the 1st time point. (**a2**): Trend series of PMLba55 (Gammaproteobacteria03170) and temperature with a LT score 0.577465 from the 1st time point (*D*=0 with *P*=0.000381, *Q*=0.017572 in approximation). (**b1**): Normalized abundance level series of PMLba7 (Alphaproteobacteria032) and DX1 (a cosine term of day length) associated from the 2nd time point. (**b2**): Trend series of PMLba7 (Alphaproteobacteria032) and DX1 (a cosine term of day length) with a LT score 0.56338 from the 2nd time point (*D*=1 with *P*=0.000598, *Q*=0.022149 in approximation)

Through studying the associations between environment factors and bacteria OTUs, we find that day length (DX1) and temperature are the main factors associated with bacteria among the 15 environment factors we select. PMLba55 (Gammaproteobacteria03170) and temperature are associated with a LT score of 0.5774 from the 1st time point (*D*=0 with *P*=0.0004, *Q*=0.0176). The abundance levels and trend series of PMLba55 and temperature are shown in Fig. 6(a1) and Fig. 6(a2), respectively. PMLba7 (Alphaproteobacteria032) and day length are associated with a LT score of 0.5633 starting from the 2nd time point (*D*=1 with *P*=0.0006, *Q*=0.0221). The abundance levels and trend series and day length are shown in Fig. 6(b1) and Fig. 6(b2), respectively.

**Fig. 7:**
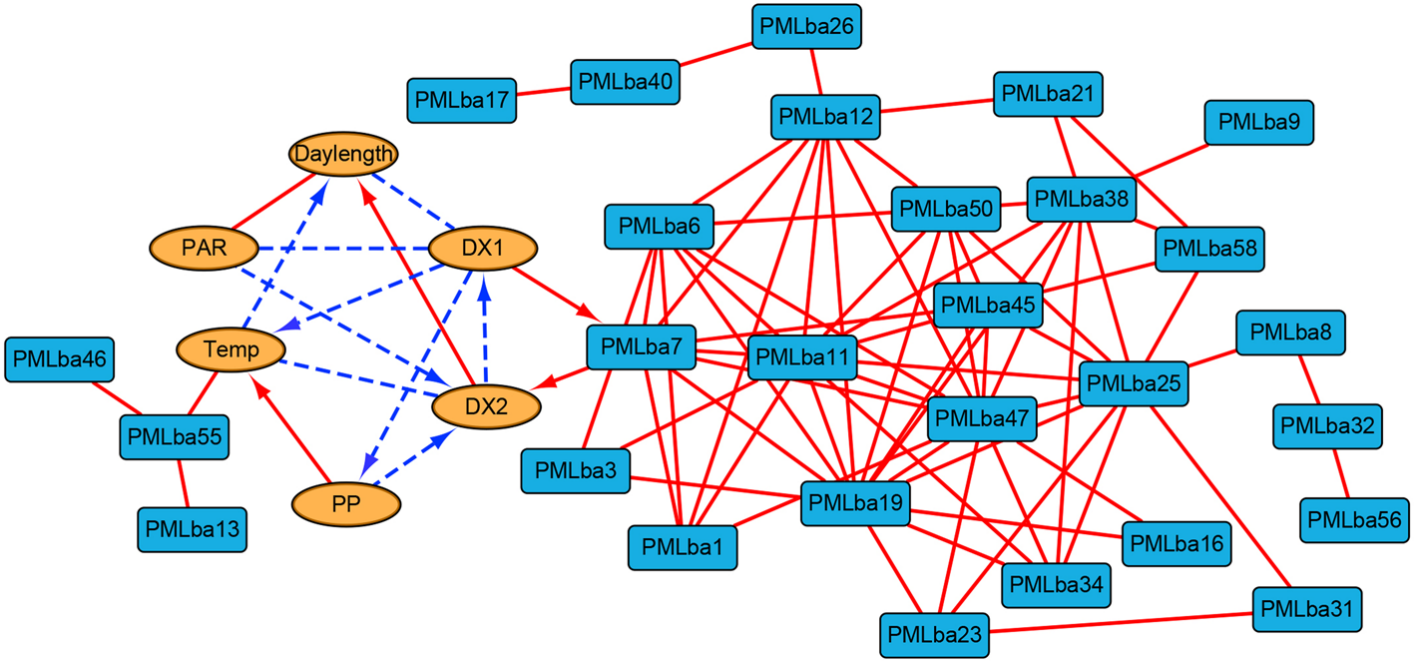
An association network generated from the PML microbial community data. Round (brown), square (blue) shaped nodes are environmental factors and bacteria, respectively. Solid (red) edges are positively associated, while dashed (blue) edges are negatively associated. Arrows indicate the time-delay direction

Overall, we show that the majority of positive associated OTU pairs are within the same phylum, while there are some associations between different phylum. Finally, we used Cytoscape to create a network from the selected PML data as shown in Fig. 7. The hubs with large number of associations are Alphaproteobacteria (PMLba1, PMLba11, PMLba26, etc.). However, the environment factors are not directly associated with Alphaproteobacteria. Most bacteria association are synchronized and delays can only be found between environment factors and PMLba7. In addition, all the associations among bacteria trend series are positive. The eLSA software package is used for analyzing the relationships between environment factors and bacteria and generating the interaction network.

## Conclusions

Many breakthroughs in high-throughput experimental technologies have made possible very large scale time-resolved omics studies (proteomics, transcriptomics, metagenomics) possible, tracking hundreds, thousands, or even tens of thousands of molecules simultaneously. Time-series data generated from these studies provide an invaluable resource to investigate the changing dynamics of biological systems. To make full use of huge size datasets, accurate and efficient statistical and computational methods are urgently needed in all levels of analysis, from accurate estimation of abundance and expression levels, to pairwise association and network analysis.

In this paper, we provide asymptotic formulae to approximate the statistical significance of local trend scores used in local trend analysis for time series data. From our simulations and real data analysis, *P*_*theo*_ is more conservative than *P*_*perm*_– a property particularly needed in many biological applications that are prone to false positive calls, such as microarray analysis [22]. However, the power of detecting the association can be low using the approximate p-values. If more accurate p-values for significant associations are desired, we suggest a “hybrid" approach: first use a relatively loose threshold on the fast approximate p-values and obtain a relatively small set of associated pairs and then slow permutation approaches are used only for this set of associated pairs to obtain more accurate p-values. This will significantly reduce the computational time yet maintain the power.

An important reason for us to embrace the approximation is its computation efficiency. As shown in Xia et al. [5], for a given type-I error, *α*, the time complexity of computing *P*_*perm*_ is *O*(*D* *M* *N*/*α*), where *D* is the delay limit, *N* is the sample number and *M* is the replicate number. With *P*_*theo*_, before any pairwise comparison, we may compute and store (*LT* score, *p*-value) pairs into a hashing table. Then, for each comparison, it only costs constant time *O*(1) to read out *P*_*theo*_ and is independent of *D*, *M*, *N* and *α*, a strongly desired feature in large scale analysis.

For instance, in metagenomics, after short read assignment and abundance estimation [34, 35], profiles of thousands of microbial OTUs are present. Before this work, pairwise local trend analysis with this number of factors was hardly tractable using permutation procedures, if not impossible. Parallel computation and hardware acceleration or additional pre-clustering and filtering approaches are required, increasing the difficulty of analysis. With the new method, researchers can quickly compute the statistical significance for all OTU pairs on desktop computers, allowing on-the-fly association network mining and analysis. Finally, We have implemented the new method in the eLSA package [5], which now provides a high-throughput pipeline for local trend analysis.

## Availability of data and materials

The eLSA software package that implements the local trend analysis and theoretical approximation is freely available for academic use from the website: http://bitbucket.org/charade/elsa. The eLSA package is a standard Python and C++ extension module that requires a Python distribution and a C++ compiling environment to install. eLSA has been extensively tested running on Ubuntu Linux machines (see the README file coming with the software for details).

The ‘CDC’, ‘MPH’ and ‘PML’ datasets are all publicly available in the supplementary of their publications [24, 32, 33]. No ethics approval was required for the study and no informed consent was required for the study, because the study involves no human and animal subjects and the study is not generating new human data. The human microbiome data analyzed in the study was published in Caporaso et al. [33] and publicly available.

## Additional file

Additional file 1
**Simulation results for** ***t*** **=0.5.** (1004 Kb)

## Abbreviations

CDC: Cell division cycle (Dataset)
eLSA: Extended local similarity analysis
FDR: False discovery rate
LS: Local similarity
LT: Local trend
LTA: Local trend analysis
MPH: Motion picture of human (Dataset)
NGS: Next generation sequencing
OTU: Operational taxonomic unit
PCC: Pearson’s correlation coefficient
PML: Polymouth Marine Laboratory (Dataset)
