# Supplementary material for: Statistical significance approximation in local trend analysis of high-throughput time-series data using the theory of Markov chains
Source: BMC Bioinformatics. 2015 Sep 21;16:301. doi: 10.1186/s12859-015-0732-8 (PMC4578688; doi:10.1186/s12859-015-0732-8)
Supplement: Additional file 1 — Simulation results for t =0.5. (1004 Kb) [file 12859_2015_732_MOESM1_ESM.pdf]

Statistical significance approximation in local trend analysis of high-throughput time-series data using the theory of Markov chains: Additional File 1

Li C. Xia[1,2], Dongmei Ai[3], Jacob A. Cram[4], Xiaoyi Liang[3], Jed A. Fuhrman[4], Fengzhu Sun[5,6*]

[1]Department of Medicine, Division of Oncology, Stanford University School of Medicine, Stanford, CA 94305-5151, USA
[2]Department of Statistics, the Wharton School, University of Pennsylvania, Philadelphia, PA 19104, USA
[3]School of Mathematics and Physics, University of Science and Technology Beijing, Beijing, 100083, China.
[4]Marine and Environmental Biology, Department of Biological Sciences, University of Southern California, Los Angeles, CA 90089-0371, USA
[5]Molecular and Computational Biology, Department of Biological Sciences, University of Southern California, Los Angeles, CA 90089-2910, USA
[6]Centre for Computational Systems Biology, Fudan University, Shanghai, China

Email: Fengzhu Sun*- fsun@usc.edu;

*Corresponding author

Additional Results

Approximating the tail probability of the LT score for the three letter alphabet case (t=0.5)

In this additional file, we compare the approximate p-value with the simulated p-value for the three letter alphabet case with $t = 0.5$. We pre-calculate $\sigma_{d^X d^Y} = \sqrt{0.92}$. Table SS1 gives the approximate tail probability (p-value) (2nd column) and the simulated probability $P(LT(0)/\sqrt{0.92n} \geq x)$ (3rd to 9th columns) for different numbers of time points when $D = 0$. Starting from $n = 20$ time points, the approximate tail probability is close to the simulated probability when the approximate p-value is less than 0.05 in the sense that the first non-zero decimal of the approximate p-value is mostly the same as that from the permutational p-value. Like the $t = 0$ case, the approximate tail probability is slightly larger than the simulated values when $D = 0$ (see Table S1) and similarly for $D = 1, 2, 3$ (see Tables S2-S4). Again, it will be slightly conservative in declaring significant associations if we use the approximate tail distribution to calculate the p-value.

We next see how p-values (P_{theo}) derived from our approximation compare to that from permutation (P_{perm}) given the same simulated time series data. As for $D = 0$, starting from $n = 20$, points in scatter plots become concentrated on the diagonal line (where P_{perm}=P_{theo}) and they become more aligned to it

as n increases (see Figure S1). This indicates an increasing rate of agreement between the approximate and permutation p-values, representing their reasonable approximation to the null distribution in spite of the inherent randomness associated with the permutation procedures. The same is true with $D = 1, 2, 3$ as the approximation become significantly closer to the permutation one when n increases. Though, when $D = 1, 2, 3$, the bias toward the upper diagonal seems more substantial and close alignment only starts at n above 30. In summary, we can see that if we are interested in statistical significance at a given type I error threshold, the approximation provides results comparable to that from permutations starting from $n = 20$ to 30.

Figures

Figure S1 - Comparison of P_{theo} and P_{perm} for simulation data $t = 0.5$

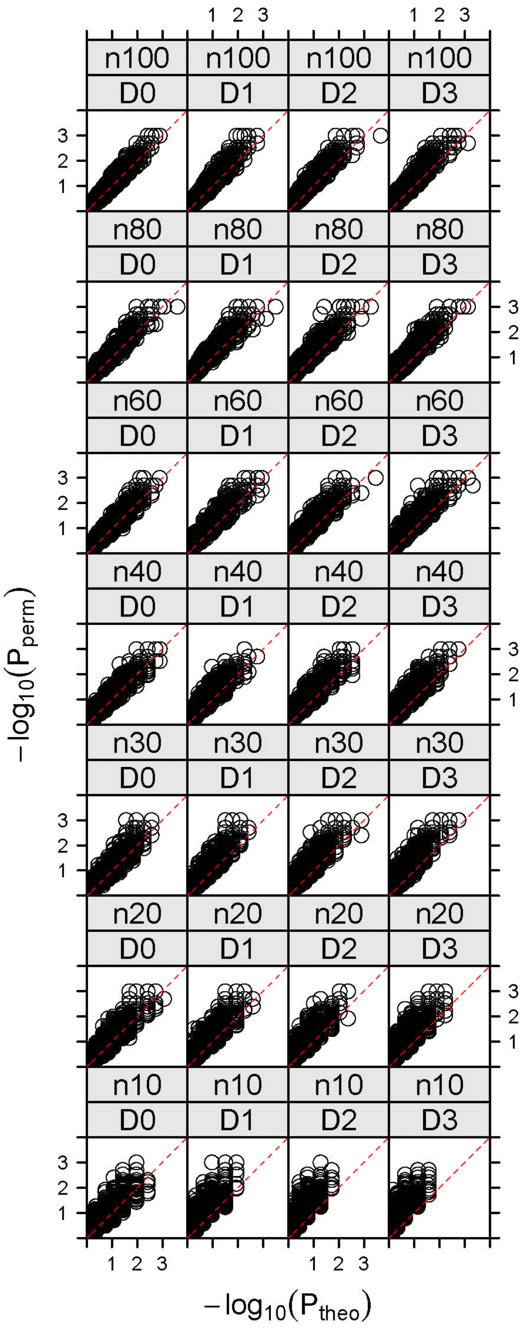

Figure S1: Local trend analysis ($t = 0.5$). The values of P_{theo} vs P_{perm} for 10,000 pairs simulated data. Columns D0 to D3 are for $D = 0, 1, 2, 3$. Rows n10 to n100 are for $n = 10, 20, 30, 40, 60, 80, 100$.

Tables

Table S1 - Approximate versus empirical tail probability ($t = 0.5, D = 0$)
Table S2 - Approximate versus empirical tail probability ($t = 0.5, D = 1$)
Table S3 - Approximate versus empirical tail probability ($t = 0.5, D = 2$)
Table S4 - Approximate versus empirical tail probability ($t = 0.5, D = 3$)

x	Approximation	The number of time points n						
		10	20	30	40	60	80	100
2.0	0.1815	0.0654	0.1124	0.1055	0.0971	0.1334	0.1136	0.1183
2.2	0.1111	0.0654	0.0581	0.0620	0.0600	0.0640	0.0834	0.0672
2.4	0.0656	0.0229	0.0270	0.0354	0.0377	0.0436	0.0443	0.0373
2.6	0.0373	0.0229	0.0133	0.0200	0.0220	0.0191	0.0225	0.0264
2.8	0.0204	0.0060	0.0056	0.0106	0.0129	0.0123	0.0106	0.0119
3.0	0.0108	0.0007	0.0056	0.0047	0.0046	0.0048	0.0065	0.0060
3.2	0.0055	0.0007	0.0030	0.0018	0.0028	0.0027	0.0026	0.0026
3.4	0.0027	0.0000	0.0012	0.0007	0.0011	0.0007	0.0010	0.0013
3.6	0.0013	0.0000	0.0004	0.0000	0.0008	0.0006	0.0006	0.0006
3.8	0.0006	0.0000	0.0000	0.0000	0.0001	0.0001	0.0001	0.0002
4.0	0.0003	0.0000	0.0000	0.0000	0.0000	0.0000	0.0000	0.0001
4.2	0.0001	0.0000	0.0000	0.0000	0.0000	0.0000	0.0000	0.0000

Table S1: Approximation for the tail probability of local trend score (LT score) versus the simulated probability $P(LT(D)/\sqrt{0.92n} \geq x)$. The approximate probability based on equation (13) is given in the 2nd column and the probability that $LT(D)/\sqrt{0.92n} \geq x$ from simulations is given in the 3rd to the 9th columns. Here, $D = 0$.

x	Approximation	The number of time points n						
		10	20	30	40	60	80	100
2.0	0.4516	0.1221	0.2359	0.2467	0.2235	0.3071	0.2604	0.2708
2.2	0.2977	0.1221	0.1263	0.1493	0.1492	0.1633	0.1992	0.1643
2.4	0.1841	0.0415	0.0627	0.0871	0.0970	0.1187	0.1137	0.0907
2.8	0.0601	0.0085	0.0128	0.0250	0.0346	0.0346	0.0255	0.0356
3.0	0.0320	0.0010	0.0128	0.0130	0.0098	0.0132	0.0170	0.0176
3.2	0.0164	0.0010	0.0047	0.0058	0.0047	0.0086	0.0087	0.0081
3.4	0.0081	0.0000	0.0013	0.0025	0.0019	0.0023	0.0037	0.0029
3.6	0.0038	0.0000	0.0005	0.0011	0.0006	0.0016	0.0027	0.0010
3.8	0.0017	0.0000	0.0001	0.0005	0.0001	0.0008	0.0011	0.0002
4.0	0.0008	0.0000	0.0001	0.0000	0.0000	0.0004	0.0006	0.0000
4.2	0.0003	0.0000	0.0000	0.0000	0.0000	0.0002	0.0001	0.0000
4.4	0.0001	0.0000	0.0000	0.0000	0.0000	0.0001	0.0000	0.0000
4.6	0.0001	0.0000	0.0000	0.0000	0.0000	0.0001	0.0000	0.0000

Table S2: Approximation for the tail probability of local trend score (LT score) versus the simulated probability $P(LT(D)/\sqrt{0.92n} \geq x)$. The approximate probability based on equation (13) is given in the 2nd column and the probability that $LT(D)/\sqrt{0.92n} \geq x$ from simulations is given in the 3rd to the 9th columns. Here, $D = 1$.

		The number of time points n						
x	Approximation	10	20	30	40	60	80	100
2.0	0.6326	0.1593	0.3176	0.3300	0.3262	0.4309	0.3709	0.3920
2.2	0.4452	0.1593	0.1806	0.2109	0.2171	0.2351	0.2828	0.2495
2.4	0.2876	0.0454	0.0902	0.1252	0.1387	0.1677	0.1582	0.1477
2.6	0.1730	0.0454	0.0393	0.0698	0.0856	0.0818	0.0813	0.1098
2.8	0.0981	0.0090	0.0161	0.0392	0.0522	0.0541	0.0389	0.0591
3.0	0.0528	0.0006	0.0161	0.0210	0.0161	0.0211	0.0266	0.0320
3.2	0.0272	0.0006	0.0055	0.0099	0.0088	0.0136	0.0105	0.0157
3.4	0.0134	0.0000	0.0014	0.0035	0.0052	0.0040	0.0037	0.0065
3.6	0.0063	0.0000	0.0004	0.0015	0.0026	0.0026	0.0023	0.0027
3.8	0.0029	0.0000	0.0000	0.0007	0.0003	0.0007	0.0006	0.0011
4.0	0.0013	0.0000	0.0000	0.0000	0.0003	0.0005	0.0002	0.0004
4.2	0.0005	0.0000	0.0000	0.0000	0.0001	0.0002	0.0000	0.0002
4.4	0.0002	0.0000	0.0000	0.0000	0.0000	0.0000	0.0000	0.0000
4.6	0.0001	0.0000	0.0000	0.0000	0.0000	0.0000	0.0000	0.0000

Table S3: Approximation for the tail probability of local trend score (LT score) versus the simulated probability $P(LT(D)/\sqrt{0.92n} \geq x)$. The approximate probability based on equation (13) is given in the 2nd column and the probability that $LT(D)/\sqrt{0.92n} \geq x$ from simulations is given in the 3rd to the 9th columns. Here, $D = 2$.

		The number of time points n						
x	Approximation	10	20	30	40	60	80	100
2.0	0.7539	0.1674	0.3886	0.4071	0.4034	0.5409	0.4769	0.4894
2.2	0.5616	0.1674	0.2163	0.2591	0.2749	0.3138	0.3722	0.3164
2.4	0.3779	0.0535	0.1071	0.1551	0.1769	0.2248	0.2137	0.1887
2.6	0.2336	0.0535	0.0478	0.0842	0.1103	0.1052	0.1112	0.1446
2.8	0.1346	0.0110	0.0204	0.0436	0.0648	0.0690	0.0511	0.0781
3.0	0.0732	0.0016	0.0204	0.0241	0.0191	0.0286	0.0343	0.0380
3.2	0.0379	0.0016	0.0079	0.0116	0.0099	0.0182	0.0130	0.0166
3.4	0.0187	0.0000	0.0018	0.0053	0.0050	0.0067	0.0059	0.0084
3.6	0.0089	0.0000	0.0009	0.0018	0.0025	0.0039	0.0034	0.0038
3.8	0.0040	0.0000	0.0000	0.0009	0.0006	0.0013	0.0014	0.0013
4.0	0.0018	0.0000	0.0000	0.0000	0.0002	0.0007	0.0006	0.0003
4.2	0.0007	0.0000	0.0000	0.0000	0.0001	0.0001	0.0003	0.0000
4.4	0.0003	0.0000	0.0000	0.0000	0.0000	0.0000	0.0001	0.0000
4.6	0.0001	0.0000	0.0000	0.0000	0.0000	0.0000	0.0000	0.0000

Table S4: Approximation for the tail probability of local trend score (LT score) versus the simulated probability $P(LT(D)/\sqrt{0.92n} \geq x)$. The approximate probability based on equation (13) is given in the 2nd column and the probability that $LT(D)/\sqrt{0.92n} \geq x$ from simulations is given in the 3rd to the 9th columns. Here, $D = 3$.
